# Daughter cell-targeted mRNAs can achieve segregation without the universal Endoplasmic Reticulum docker She2p

**DOI:** 10.17912/micropub.biology.000458

**Published:** 2021-09-13

**Authors:** Kseniya Samardak, María Moriel-Carretero

**Affiliations:** 1 Centre de Recherche en Biologie cellulaire de Montpellier (CRBM), Université de Montpellier, Centre National de la Recherche Scientifique, 34293 Montpellier CEDEX 05, France

## Abstract

The establishment of cell polarity in eukaryotes involves the asymmetric distribution of messenger RNAs (mRNAs). In *Saccharomyces cerevisiae*, establishment of the cell polarity that gives rise to mother and daughter cells concurs with the selective targeting of more than 30 mRNAs toward the bud tip. Different mRNAs are segregated at different cell cycle stages, namely early during S phase, in a process dependent on anchoring to the endoplasmic reticulum (ER), or later in G_2_ or mitosis, in an ER-independent manner. In spite of this difference, this transport requires in all cases the Myo4p motor and its interaction with actin, the adaptor protein She3p and a third, RNA-binding protein docking this complex at the mRNA itself. This protein is universally considered to be She2p. Yet, the majority of mRNAs whose segregation was shown to be She2p-dependent are not S-phase segregated ones. In other processes aimed at establishing polarity, such as during pheromone-stimulated G_1_ arrest, the coupling of mRNAs to the ER during their transport is She2p-independent. We have therefore asked if the segregation to the bud of a model S-phase-specific mRNA, *EAR1*, is dependent on She2p or not. We report that a modest yet consistent percentage of *EAR1* segregating particles achieves polarization without She2p. Our data invite to a re-evaluation of the absolute necessity for She2p for daughter cell-targeted mRNAs distribution.

**Figure 1. Evaluation of the dependency on She2p for the delivery to the bud of the S-phase-segregated mRNA EAR1:**
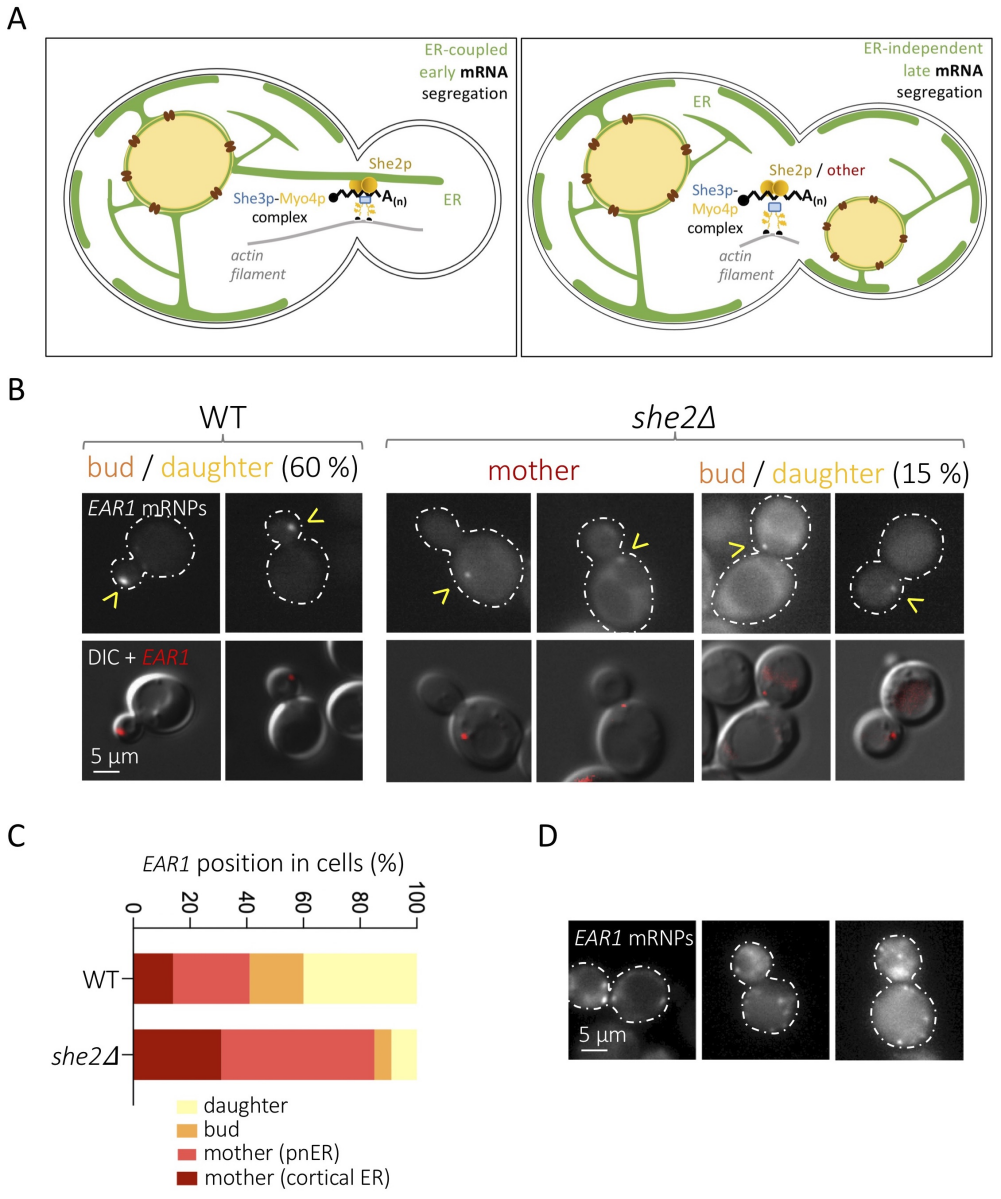
**(A)** Scheme depicting the two presently defined pathways to deliver polarized mRNAs (in black) to the bud/daughter in *S. cerevisiae*, both thought to be She2p-dependent (Fundakowski *et al.* 2012). **Left:** the mRNAs expressed in S phase are anchored via She2p to the segregating endoplasmic reticulum (ER), which advances towards the bud. **Right:** the mRNAs expressed in G_2_ and mitosis migrate towards the daughter cell in an ER-independent manner. In both scenarios, She2p is thought to couple the mRNAs to the She3p adaptor and the Myo4p motor, which promotes migration thanks to its interaction with actin filaments. **(B)** Representative images (two per condition) of cells expressing *EAR1* recognized by MS2-mCherry (yellow arrowheads) in wild-type cells (left) and in the *she2Δ* mutant (right). The most frequent localization profile in WT cells, that is, *EAR1*-MS2-mCherry particles in daughter cells, and both in-the-mother and in-the-daughter *EAR1*-MS2-mCherry particles in *she2Δ* are shown. Differential Interference Contrast (DIC) and DsRed fluorescent channels (on which the cell contour has been over-imposed as dashed lines) are shown. **(C)** Quantification of *EAR1* mRNPs distribution in the indicated strains. Plots represent the values obtained in 4 to 5 independent experiments, where at least 30 foci per experiment were counted. It was exceptional to detect more than one *EAR1* fluorescent particle per cell. At least 350 cells were scored per experiment. The foci in the mother cell were sub-classified as near the cortical ER (periphery) or the perinuclear one (pnER). To assess this latter pattern, the physical proximity to nuclear DNA was estimated by the simultaneous staining with DAPI, not displayed. **(D)** Example of three WT cells in which MS2-mCherry-associated signals appear as multiple, randomly distributed foci. This pattern was absent in *she2Δ* cells and rare (eventually found in about ten cells per experiment) in WT ones.

## Description

The establishment of cell polarity in eukaryotes involves the asymmetric distribution of messenger RNAs (mRNAs). In *Saccharomyces cerevisiae*, polarization leads to the budding of daughter cells, and more than 30 mRNAs subjected to selective targeting toward the bud tip have been characterized (Shepard *et al.* 2003; Aronov *et al.* 2007). Different mRNAs are segregated at different cell cycle stages, which is mostly dictated by their expression profiles. This way, mRNAs such as *WSC2*, *IST2* or *EAR1* are delivered during S / G_2_ phases, when the bud starts to emerge, while others, such as *ASH1*, are dispatched late during mitosis (Shepard *et al.* 2003; Fundakowski *et al.* 2012). Importantly, segregation of “early” mRNAs is coupled to, and dependent on, the simultaneous segregation of the endoplasmic reticulum (ER) toward the daughter cell (Figure 1A, left panel), while late-segregating mRNAs are not dependent on ER inheritance (Figure 1A, right panel) (Fundakowski *et al.* 2012). In both cases though, the trafficking of these mRNAs requests the myosin Myo4p, which promotes transport along actin filaments, and the adaptor protein She3p (Münchow *et al.* 1999) (Figure 1A). Using the mitosis-specific *ASH1* mRNA as a model, a third, RNA-binding protein, She2p, was implicated as part of this “locasome” complex, its task being that of bridging the mRNA to the motor (Münchow *et al.* 1999; Bohl *et al.* 2000; Long *et al.* 2000; Takizawa and Vale 2000). Both the classical model *ASH1* mRNA and 22 newly identified polarized mRNAs were found to be dependent on She2p for segregation (Bertrand *et al.* 1998; Shepard *et al.* 2003), and additional, asymmetrically distributed mRNAs, involved in polarity and exocytosis, were seen to co-fractionate with She2p (Aronov *et al.* 2007). Together, this has led to the general view that She2p is the universal factor docking mRNAs destined for the daughter cell at the Myo4p-She3p transporting complex. What is more, in the case of S-phase-segregating mRNAs, She2p is proposed to couple mRNA distribution and ER inheritance through its ER membrane-binding properties (Genz *et al.* 2013) (Figure 1A, left panel).

We realized that, of the 22 mRNAs whose asymmetric distribution has been demonstrated to rely on She2p, only 2 (*DNM1* and *WSC2*) were early, S-phase-segregated (Shepard *et al.* 2003). Other early segregating mRNAs were shown to depend on She2p to associate to the ER, yet whether this is necessary to reach the bud was not evaluated (Aronov *et al.* 2007). We have therefore assessed, for the first time, the *bona-fide* “early segregating” mRNA *EAR1*, as defined by its dependence on ER inheritance (Fundakowski *et al.* 2012), for its dependency (or not) on She2p in order to be polarized. We transformed wild type (WT) and *she2∆* *S. cerevisiae* cells with two vectors: one expressing the mCherry fluorophore fused to the coding sequence for the single-stranded RNA phage capsid protein MS2, and another plasmid expressing, from its own promoter, a modified *EAR1* bearing 12 stem loops recognized by the MS2 protein (Bertrand *et al.* 1998). Cells growing exponentially in minimal medium selective for both vectors were imaged to observe the position of MS2-revealed *EAR1* mRNA particles (mRNPs) (Figure 1B). We found that the absence of She2p substantially limits *EAR1* delivery to the daughter cell, yet does not abolish it (Figure 1B,C). In particular, we observed that, compared to the 60% of MS2-defined *EAR1* foci reaching the bud and the daughter in the WT in asynchronous cultures, only 15% did in *she2∆* cells (Figure 1B,C). This is relevant, because a 15% delivery success is classified in the literature as weak yet existent targeting (Shepard *et al.* 2003). Further, defective mRNA particle delivery of concerned mRNAs in *she2∆* cells has been described to concur with abundant, randomly distributed, tiny signals (Shepard *et al.*2003), something we never observed for *EAR1* in *she2∆* cells, and seldom in WT ones (Figure 1D).

As a whole, our data suggest that the segregation of some daughter-targeted mRNAs may occur in the absence of She2p. This remains a novelty because, up-to-date, delivery events are reported to be She2p-dependent (Bertrand *et al.* 1998; Shepard *et al.* 2003). It could be that additional factors, yet to be identified, are in charge of a subpopulation of these mRNAs (Figure 1A, right panel, “other”). It is indeed tempting to imagine that some bud-targeted mRNAs are not linked to the Myo4p-She3p complex and actin filaments through She2p. There is evidence for the existence of alternative dockers, as is the case for Scp160p, which fulfils this function during the polarized delivery of mRNAs to the shmoo tip in G_1_ phase in response to sexual pheromones (Gelin-Licht *et al.* 2012). But it could also be argued that the events in which we detected *EAR1* in the daughter cell are due to passive diffusion. Even if random MS2 spots have been reported to distribute as multiple, small, disordered foci (Bertrand *et al.* 1998), which is not what we detect (Figure 1D), this is not enough to dismiss the possibility that *EAR1* particles manage to reach the daughter cell passively. Since She2p is needed to anchor the targeted mRNA to the early segregating ER, it is likely that *EAR1* She2p-independent distribution to the daughter occurs later in the cell cycle thanks to the prolonged window of time before division. Last, it could also be argued that the MS2-mCherry tagging supports artefactual association of molecules, thus favouring their segregation. Although possible, this is unlikely, as MS2-driven and *in-situ* hybridization-mediated mRNAs detection proves comparable (Schmid *et al.* 2006). In all instances, our work thus establishes a precedent justifying the careful assessment of other early bud-segregated mRNAs for their (in)dependence on She2p, or on alternative dockers.

## Methods

*Saccharomyces cerevisiae* cells were grown at 25°C in selective YNB liquid medium supplemented with 2% glucose without histidine and uracil to ensure plasmid maintenance. All experiments were performed with exponentially growing cells. For microscopy analyses, 1 mL of the culture of interest was centrifuged; then, the supernatant was thrown away and the pellet was resuspended in the remaining 50 μL. Next, 3 μL of this cell suspension was directly mounted on a coverslip for immediate imaging of MS2-mCherry signals. Fluorescent signals were detected using the adequate wavelength and acquired with a Zeiss Axioimager Z2 microscope and Metamorph software. Subsequent image visualization and analysis were performed with Image J v2.0.0-rc-69/1.52i. The determination of the sub-cellular localization of *EAR1* signals in all cells displaying any was done through visual inspection by the experimenter. GraphPad Prism was used to plot the results.

## Reagents

The wild-type strain (MM-35) is a W303 strain corrected for the *RAD5* gene and the *she2∆* strain (MM-302) was built by classical gene disruption using the G418 resistance cassette *kanMX6*. Plasmids RJP1815 (pYCplac33-*EAR1*-12MS2-*URA3*) and RJP1889 (pCP-MS2-5xmCherry-*HIS3)* were a kind gift from Pr. Ralf-Peter Jansen, Tübingen University. We also used DAPI (D9542, Sigma-Aldrich).
